# An Ultrahigh Sensitive Microwave Microfluidic System for Fast and Continuous Measurements of Liquid Solution Concentrations

**DOI:** 10.3390/s21175816

**Published:** 2021-08-29

**Authors:** Piotr Słobodzian, Krzysztof Szostak, Katarzyna Skowronek, Laura Jasińska, Karol Malecha

**Affiliations:** 1 Department of Telecommunications and Teleinformatics, Faculty of Electronics, Wrocław University of Science and Technology, 50-370 Wrocław, Poland; piotr.slobodzian@pwr.edu.pl (P.S.); krzysztof.szostak@pwr.edu.pl (K.S.); 2 Faculty of Pure and Applied Mathematics, Wrocław University of Science and Technology, 50-370 Wrocław, Poland; 226245@student.pwr.edu.pl; 3 Department of Microsystems, Faculty of Microsystem Electronics and Photonics, Wrocław University of Science and Technology, 50-370 Wrocław, Poland; laura.jasinska@pwr.edu.pl

**Keywords:** concentration measurement, microwave interferometry, microwave-microfluidic system, LTCC sensor, microwave sensor

## Abstract

In this paper, we describe a low-cost microwave microfluidic system of ultrahigh sensitivity for detecting small changes in the concentration of polar solutions (liquid dielectrics) in the 2.4 GHz ISM band. Its principle of operation is based on microwave interferometry, which is implemented using planar microstrip lines and integrated microwave components. The key features of this system include small solution intake (<200 µL per measurement), short time of measurement (ca. 20 ms), ultrahigh sensitivity of concentration changes (up to 55 dB/%), and low error of measurement (below 0.1%). The ultrahigh sensitivity was proven experimentally by measurements of the fat content of milk. In addition, it is a user-friendly system due to an effortless and fast calibration procedure. Moreover, it can be made relatively compact (<20 cm^2^) and features low power consumption (200 mW). Thus, the proposed system is perfect for industrial applications, especially for highly integrated lab-on-chip devices.

## 1. Introduction

The need for precise, cost-efficient and real-time monitoring of minimal changes of many substances is crucial for many industries and life sciences, such as food fraud detection [1], automated drug discovery [2], organ-on-a-chip engineering [3], and dangerous liquid measurements [4]. It is also essential for advanced RADAR and communication technology solutions that use liquid dielectrics [5,6,7].

There are many methods designed to monitor the composition of liquid solutions. It seems that fluorescence spectroscopy methods are the most widely held, mainly in biomedical applications [8]. Nevertheless, those methods possess several substantial disadvantages in industrial applications, such as the need for special preparation of samples or the use of transparent media and photon detectors with limited spectral absorption. Therefore, they generally destroy samples during the measurement process. Furthermore, those methods do not fit the industry environment perfectly since they require expensive and fragile refractometers. Advances in nondestructive techniques based on contactless microwave spectroscopy have recently been the subjects of comprehensive studies. Their scope of application is expanding, as described extensively in [4].

A comprehensive overview of microwave sensors for liquid solutions is given in [9,10]. The principle of operation of most microwave sensors is connected with different microfluidic technologies [11,12]. One of these technologies is LTCC (low temperature cofired ceramics), which has been successfully used to manufacture microwave devices with an embedded microfluidic channel to monitor many chemical compounds [13]. Due to the excellent chemical resistivity of LTCC materials and their good microwave properties, this technology can provide a reliable means of measuring food nutrition content, quality, and spoiling process, which is in high demand [13,14]. Some trials have already been performed in this field, including microwave measurements of the quality and composition of products such as meat, fruits, and vegetables using the coaxial probe [15] or milk using a five-port reflectometer [16]. Unfortunately, all those methods suffer from insufficient sensitivity. In addition, such methods require using an expensive vector network analyzer (VNA) or low-cost hardware that involves a highly complicated calibration.

In this paper, we describe a simple microwave microfluidic interferometric sensor of extremely high sensitivity. This type of interferometric sensor is currently popular and well established [17,18,19,20,21,22]. Our solution is somewhat similar to the system described in [20], which contains a tunable phase shifter and a tunable attenuator in the test channel to obtain deep interference nulls. However, those two elements were moved to the reference channel in our system, and the test channel was equipped with an additional tunable attenuator. Such an approach allowed the system to be better balanced than the system proposed in [20]. The phase shifter in the reference channel directly compensates for a phase delay introduced by the test channel. The two attenuators enable more precise compensation/equalization of both channels’ reflection and insertion loss. These modifications provide a better balance of the channels and makes the system extremely sensitive. Our amendment also assures a broader range of operation of the system. Other unique features of the proposed system include the following: (1) easy calibration of freely chosen operating points to obtain high sensitivity in the required range of operation and to simultaneously enable reliable measurements of a wide range of liquid solution concentrations and (2) continuous (under control) monitoring of the concentration of liquid solutions using low-power and low-cost MCU-controlled MMIC devices (no need for spectrum or network analyzers). The performance and usefulness of the proposed system have been verified using measurements of milk with different fat contents. This paper is organized as follows: First, the theory behind the sensor’s principle of operation is explained; secondly, the design, simulation, and fabrication technology of the sensor are described; finally, experimental validation of the sensor’s demonstrator is presented, including a methodic analysis of its performance.

## 2. Materials and Methods

Measurement methods based on planar transmission lines (broadband methods) [23] could be good candidates for industrial applications, but they suffer from low sensitivity and accuracy. The resonant (narrowband) methods [24] are devoid of the disadvantages mentioned above. However, there are difficulties with tuning the sensing resonator when required. Significant improvement in the sensitivity of microwave sensors can be achieved with differential (interferometric) methods. In [20], a broadband coplanar interferometric sensor for testing liquid solution was presented. It could monitor 2-propanol concentration in deionized water as low as 0.01 mol fraction. Unfortunately, these measurements required a high-priced vector network analyzer (VNA) and advanced data processing. Another design was based on the unique properties of metamaterials [21], which allowed the permittivity of the liquid chemical to be detected with an RMS error <1.5%. In turn, in [9], the electromagnetic bandgap (EBG) structure within a microstrip line was used to obtain a relatively high sensitivity of 0.54 degrees in the measured phase shift per percentage of toluene concentration.

Moreover, in [22], a narrowband differential, coplanar sensor operating at 40 GHz was proposed with a sensitivity up to 2.5 dB/%$\Delta \varepsilon_{r,eff}$ for the concentration of ethanol. However, as mentioned in the introduction, such methods are characterized by the need for using high-cost laboratory equipment or a complicated calibration process. Such difficulties have been eliminated in the sensor system presented in this paper.

### 2.1. The Principle of Operation

**Figure 1:**
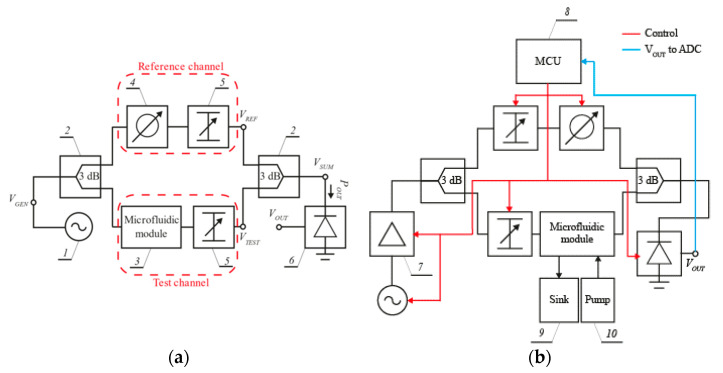
Block schemes of the proposed interferometric system: (**a**) Operating principle; (**b**) validation set-up. 1—VCO, 2—power splitter/combiner, 3—LTCC sensor module with a microfluidic channel, 4—adjustable phase shifter, 5—adjustable attenuator, 6—power detector, 7—power amplifier, 8—MCU controller, 9—sink, 10—pump.

The principle of operation and validation set-up of the proposed microwave microfluidic system are shown in Figure 1a,b, respectively.

The system consists of a signal generator, a power splitter and combiner, two transmission channels (the test and reference one), and a microwave power detector. The liquid sample under test is introduced into a microwave microfluidic module within the test channel, containing a piece of the microstrip line. This produces a change in the phase shift of the signal transmitted through this channel. In turn, the reference channel contains a tunable phase shifter used to balance the phase shift of both channels during the calibration procedure. Finally, both the reference and test channels include tunable attenuators that balance the amplitude of signals passing through these channels.

The system operates as follows: The voltage *V_GEN_* generated by the source is equally divided by the splitter. The resulting signals are fed into the test and the reference channel and produce voltages $V_{REF}$ and $V_{TEST}$ at their ends, respectively. These voltages are related to currents *I_REF_* and *I_TEST_*, which are added up by the power combiner and produce the output power ($P_{OUT}$) at the power detector. The reading of the detector depends on the difference in the phase shift Δφ between the two channels. Significantly, the phase shift of the reference channel can be adjusted during the calibration process so that both currents are out of phase and cancel each other out (provided their amplitudes are equal). 

The amplitudes can be equalized using adjustable attenuators. All these adjustments allow a selection of the so-called operating point of the system (the lowest possible $P_{OUT}$) for a reference sample of liquid solution. When the concentration of this solution is changing, the phase shift introduced by the test channel is changing simultaneously. In turn, it results in a change of Δφ. The relation between $P_{OUT}$ and Δφ can be illustrated using the so-called detection/sensing curve of the system. The phase shift introduced by the test channel ($\varphi_{TEST}$) is proportional (to an additive constant) to the length and the wavenumber of the microstrip line, as described below [25]:

(1) $$\varphi_{TEST}\sim L\cdot 2\pi f\;\sqrt{\mu_{eff}\epsilon_{eff}}$$

where f is the frequency of microwave signals used in the system, L is the length of the transmission line, and $\epsilon_{eff}$ and $\mu_{eff}$ are the effective permittivity and permeability of the medium in this transmission line, respectively. Any variation in the concentration of solution inside the microfluidic channel produces a change of $\epsilon_{eff}$ and $\mu_{eff}$. It results in a variation of the phase shift introduced by the microfluidic module. This measurement method requires calibration, which maps particular values of the concentration to the output power $P_{OUT}$.

### 2.2. The Calibration Procedure

The calibration of the proposed system is simple. It uses the well-established calibration/standard curve [26], where measurements compare a sample of unknown concentration to a set of standard samples of known concentration. Thus, the calibration itself consists of assigning typical values of the concentration to values of the output quantity of the system ($P_{OUT}$ in our case).

The system’s calibration is made by adjusting the phase shifter and both attenuators. The calibration consists of two steps. First, an operating point of the system is selected using a standard sample of a specific concentration. Then, the system is adjusted so that the output power $P_{OUT}$ is minimal. Because of the limited sensitivity of the power sensor and the finite set of discrete adjustment possibilities of attenuators and the phase shifter, minimal $P_{OUT}$ is found within a discrete space, spanned by three discrete variables, employing a simple (brute force) searching algorithm. In this way, the best possible set of adjustments is determined. Such a set does not have to necessarily assure the global minimum of $P_{OUT}$, but the obtained value should be close to it. During the adjustment procedure, several different sets of adjustments may be found that provide a very similar level of $P_{OUT}$. In this case, the set that minimizes the insertion loss of the system should be selected. In this way, a higher S/N (signal-to-noise) ratio is achieved at the power detector. 

The error involved in this process does not significantly affect the overall system performance and can be neglected. In the second step, a series of measurements are taken for a set of standard samples to determine the system’s standard curve. The obtained values are stored in a two-column matrix or chart form. The adjustment of the system is made only in the first step. The second step consists of power reading only. Once the system is calibrated, regular measurements can be done for any concentration of a sample solution within the limits of the standard curve.

## 3. Demonstrator of the System

### 3.1. Design of Microwave Microfluidic Modules

**Figure 2:**
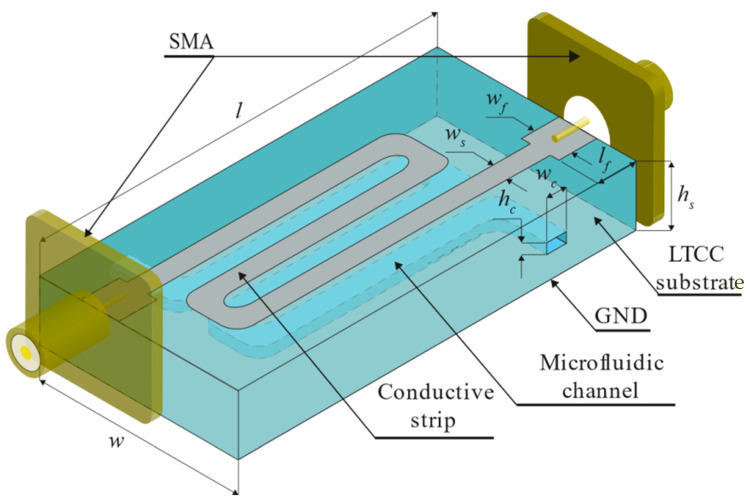
Illustrative visualization of the LTCC microwave microfluidic module with a long (meandered) microfluidic channel.

The design involves a microfluidic system and a microstrip transmission line with the 50 Ω standard for all microwave components. On the basis of our experience with LTCC materials in different microwave sensor applications (see, e.g., [13]), we chose the DuPont 951 ceramic system with green tapes of 116 µm and 254 µm in thickness (Dk=7.8, tgδ=0.003, @ 2.45 GHz and 5.12 GHz, values taken from the previous work of [27]). The next step was to determine the width and thickness of the microchannel and microstrip line. Since a liquid solution should interact with EM fields effectively, the microchannel was embedded, as illustrated in Figure 2.

**Figure 3:**
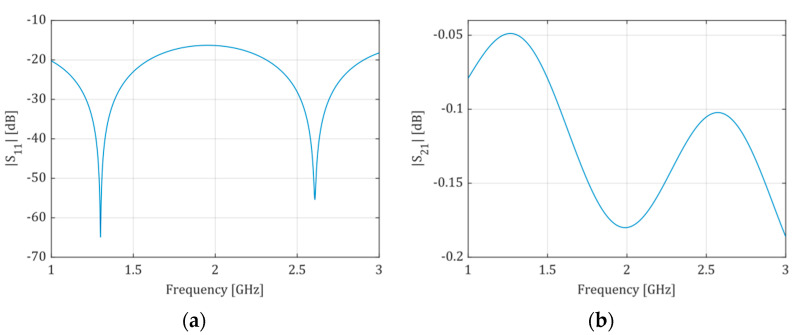
Scattering parameters obtained from simulation of the short-channel LTCC module: (**a**)—magnitude of $S_{11}$ ($S_{22}$), (**b**)—magnitude of $S_{21}$ ($S_{12}$). The structure is electrically symmetric.

**Figure 4:**
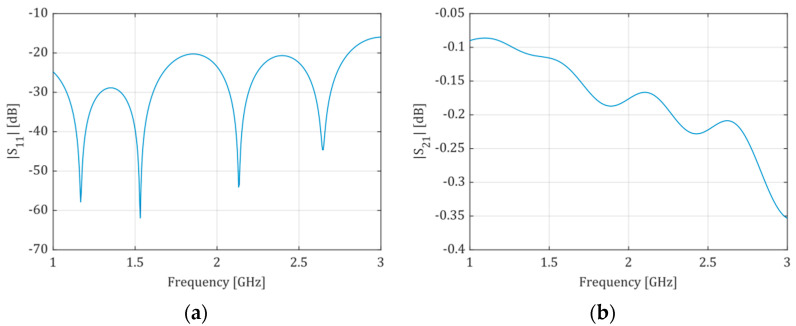
Scattering parameters obtained from simulation of the long-channel LTCC module: (**a**)—magnitude of $S_{11}$ ($S_{22}$), (**b**)—magnitude of $S_{21}$ ($S_{12}$). The structure is electrically symmetric.

The microchannel width should be enough only to enclose the fringing field under the conductive strip. This approach allows the volume of liquid inside the microchannel to be as small as possible. In turn, the width of the conductive strip depends on the effective dielectric constant of the substrate. Therefore, it should be “matched” to the liquid solution (dielectric) under test to minimize microwave power loss. Since we decided to test the system using milk samples, the microstrip line was “matched” to deionized water (Dk=78.4, taken from the CST Studio Suite Materials Library). However, both ends of the line are wider over the length of 10 mm (see, $l_{f}$), where there is no microchannel underneath. These wider parts are matched to 50 Ω using Dk of the ceramic material only, and their width is $w_{f}=1.73$ mm. The frequency range from 2.3 to 2.5 GHz was selected (ISM band). The microchannel was designed to be two times wider than the microstrip line ($w_{c}=2w_{s}$). In turn, the height $h_{c}$ of the channel was chosen to minimize all geometrical deformations during the LTCC technological process. The final height $h_{c}$ resulted from the thickness of the stack of four layers of the green tape. Two versions of the microfluidic system were designed, i.e., a short one with a straight channel and a longer, meandered one, to assess the system’s sensitivity in terms of the length L of the microstrip line. The microstrip line was terminated at both ends with the SMA connector (see Figure 2). All geometric dimensions of the two modules were optimized using the full-wave electromagnetic simulator CST Studio Suite to ensure the best impedance matching of the microstrip line in the given frequency range. Sample results of simulations for the LTCC module with a short and long microfluidic channel are presented in Figure 3 and Figure 4, respectively. The resultant dimensions are summarized in Table 1.

### 3.2. Fabrication of the Microwave Microfluidic Modules

**Figure 5:**
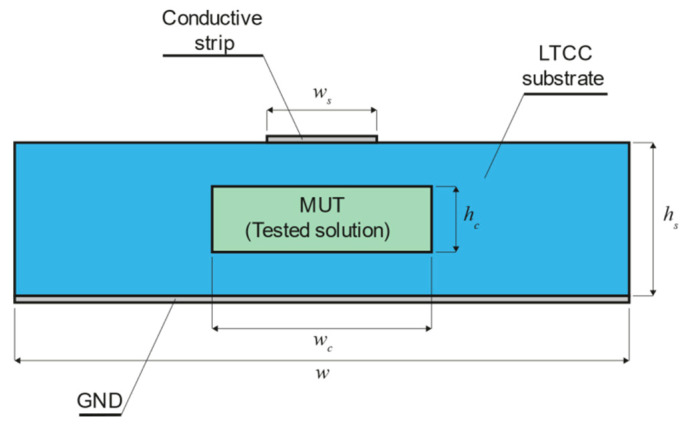
The cross-section of the sensing structure with the microfluidic channel embedded inside the substrate of the microstrip line.

All designed microwave microfluidic modules were fabricated using the standard LTCC technology. The structure of each module consists of eight layers of the DuPont 951 ceramic foil. In the first step, a rectangular shape of all layers and a shape of the microchannel was formed using the Proto Laser U (Nd:YAG, λ=355 nm) laser system. In the next step, the conductive strip was made using the screen-printing method (screen printer Aurel VS 1520A). The silver paste ESL903A with ρ≤2 mΩ/□ was used. Then, the prepared layers were piled and laminated using an isostatic press (for 10 min at the temperature of 70 °C and pressure of 3 MPa). In the next step, the laminated structure was cofired in the Nabertherm HTC 03/16 chamber furnace. Two-step temperature profiles ($T_{1}=450\;{}^{\circ}C$ and $T_{2}=880\;{}^{\circ}C$) in the air atmosphere were used. After that, the bottom surface of the module was covered with the solderable silver conductive paste ESL903D (ρ≤2 mΩ/□) using a screen-printing process, and then post-fired in the chamber furnace ($T_{max}=850\;{}^{\circ}C$). In the last step, an inlet and outlet steel capillary were attached to the structure, and two SMA edge connectors were soldered at both ends of the microstrip line. The cross-section of the structure is shown in Figure 5.

### 3.3. Setting Up and Testing the System

**Figure 6:**
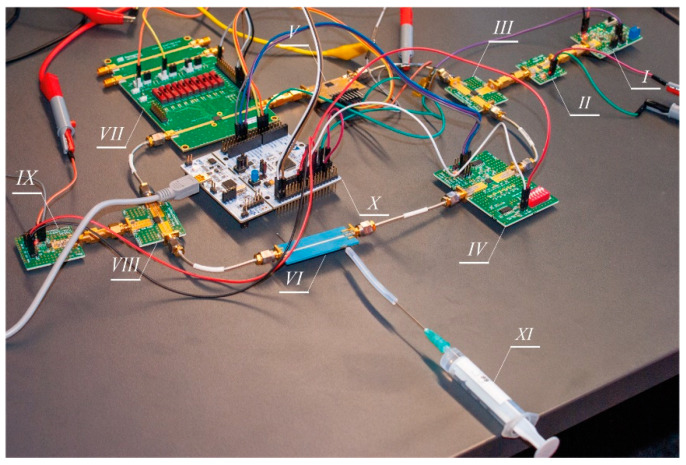
Photography of the test bench used to validate the described microwave microfluidic system for measurements of liquid solution concentrations.

To test the performance of the proposed system, a computer-controlled demonstrator, based on the scheme shown in Figure 1b, was set up. A photo of this demonstrator is shown in Figure 6.

The system consists of several off-the-shelf monolithic microwave integrated circuits (MMIC), which are digitally controlled. The test signal was generated using a MAX2750 voltage-controlled oscillator (I) with the output power of −3 dBm in the frequency range from 2.3 to 2.5 GHz. Then, to improve the quality of the detection process and increase its dynamic range, the signal was amplified by 20 dB using an MNA-6A+ low noise amplifier (II). Next, it was split in two using a 2-way 3 dB power splitter/combiner BP2U+ (III and VIII) to feed the test and reference channel, respectively. The equalization of power attenuation and phase shift of the test and reference channel at the system’s operating point was obtained using two attenuators and one phase shifter. The test channel digital attenuator DAT-31R5A-SP+ (IV) was used with an adjustment range of 0–31.5 dB and a 0.5 dB step. In turn, in the reference channel, the following MMICs were used: a digital attenuator SKY12343-364LF (V) with an adjustment range of 0–31.75 dB and 0.25 dB step, and a programmable phase shifter PE44820B-X (VII) with an adjustment range of 0–360° and 1.4° in step. The power combiner (VIII) output power was measured using a power detector LT5538 (IX) with a typical dynamic range of 75 dB, starting at −72 dBm. The output signal of this detector is an analog one, and the measured power is converted to a DC voltage signal $V_{OUT}$. Since the system is based on the standard curve, no prior calibration of the detector is required. The liquid sample under test was pumped into the microfluidic module with a small syringe (XI). A volume of not more than 200 µL of the solution was required to fill the microchannel (long channel). The system was controlled by a cheap microcontroller (X) development board NUCLEO-F072RB (ARM Cortex-M0) and an STM32F072 microcontroller was used. It features an onboard 12-bit analog-to-digital converter (ADC) with hardware calibration and a 12-bit digital-to-analog converter (DAC). The ADC was used to read voltage $V_{OUT}$ at the output of the power detector (IX). In turn, the DAC was used to control the operating frequency of the VCO source (I). Finally, a simple PC application was designed to communicate with the MCU via the USB-UART communication port to collect and process all measured data.

## 4. Results

To demonstrate the proposed system’s performance and present some possible applications, the fat content of milk was tested. The choice of test liquid was dictated by the many products with different fat contents, mainly in the food industry. Off-the-shelf products were used only (a good liquid dielectric source of different parameters) to demonstrate the calibration and measurement process.

The system’s calibration was performed for two different operating points, i.e., for the fat content of milk equal to 0% and 3.9%, respectively. In terms of the fat content, the first operating point was chosen to test the broadband performance of the system. The second operating point was selected to achieve a high resolution of the system for the low fat content of milk (<4%). In both cases, the system was adjusted to reach the minimum power reading value. After each of the calibrations, the standard curve was determined. Each sample of milk with different fat content was loaded into a separate syringe with a hermetic plug. After measuring each sample, the microchannel was cleaned with DI water and ethanol and prepared for reuse. The power readings (in dB) were mapped to the standard samples’ fat content (in %) during the calibration process. All described measurements were made for the LTCC module with a short and long microchannel at a single frequency of 2.4 GHz.

**Figure 7:**
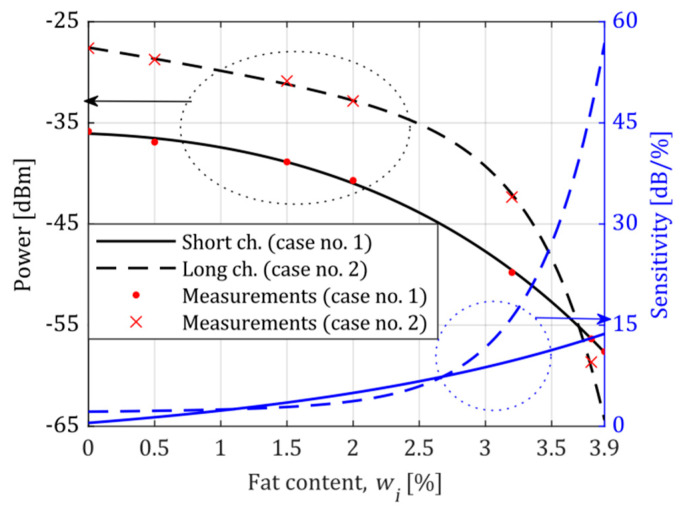
The standard curve and sensitivity curve for the fat content of milk. The system’s operating point was set at 3.9% of the milk fat. (Black curves denote power, and sensitivity is represented by blue curves, similarly to the axis colours. To distinguish measurements in the short channel “.” marker is used, whereas long channel measurements are marked by “x”).

**Figure 8:**
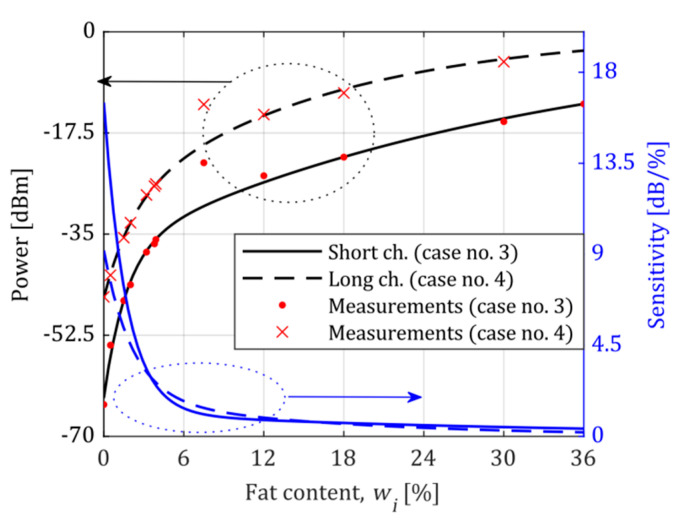
The standard curve and sensitivity curve for the fat content of milk. The system’s operating point was set at 0% of the milk fat.

A series of calibration measurements were performed to determine the system’s precision, reliability, and sensitivity. The obtained averaged standard curves for both operating points, for the short and long microchannel, are shown in Figure 7 and Figure 8.

As one can see, the system has the highest sensitivity near the operating point, which was freely selected. There is also an apparent increase in sensitivity when a longer microchannel is used. The shape of all standard curves and the related sensitivity curves are discussed in the next section.

According to the preparations mentioned above, the measurement of any milk sample is straightforward. Therefore, it suffices to feed the microfluidic module with this sample, and then read the power level at the power detector and use the standard curve to determine the fat content of milk.

## 5. Discussion

In general, the performance of any measuring and sensing instrument can be characterized by a set of parameters. The key ones are range and dynamic range, sensitivity, resolution, precision, and accuracy [28,29].

The range and dynamic range are strictly connected with the standard curve. Once the standard curve is determined, the range of permitted values of the measured parameters is readable. Hence, for the operating point set at 3.9% of milk fat, the range of fat content extends from 0% to 3.9%. In turn, for the operating point set at 0%, the range of fat content extends from 0% to 36%. Regarding the dynamic range of a measuring instrument, this parameter is typically determined in relation to the accuracy or precision of this instrument. However, in practice, this is a matter of agreement or convention and therefore, no value was assigned to this parameter.

The sensitivity of a measuring instrument is defined as the slope of the standard curve [28]. All measured standard curves obtained for Cases 1 to 4 were approximated by a two-term exponential function to determine the sensitivity. This approximation had the following form:

(2) $$P\left(w_{i}\right)=\overset{2}{\underset{k=1}{\sum }}a_{k}\cdot e^{b_{k}w_{i}}$$

where $P\left(w_{i}\right)$ designates measured power for a given standard content $w_{i}$. The approximation results are shown in Figure 7 and Figure 8 as black lines. The standard for 7.5% of milk fat (condensed milk, Figure 8) was excluded from the approximation since it diverged from the rest of the samples. A similar effect was observed and reported in [16], which probably arises from the intrinsic characteristics of condensed milk.

According to the analytical description of the standard curve, the sensitivity curve was expressed using a derivative:

(3) $$S\left(w_{i}\right)=\frac{dP\left(w_{i}\right)}{dw_{i}}=\overset{2}{\underset{k=1}{\sum }}a_{k}b_{k}\cdot e^{b_{k}w_{i}}$$

with the unit of (dB/%). The obtained sensitivity curves are shown in Figure 7 and Figure 8 as blue lines. As we can see, sensitivity reaches the highest level at the system’s operating point. The measurement of the fat content of milk shows that proper selection of the operating point of the system and the length of the microchannel can result in ultrahigh sensitivity, as shown in Figure 7 (Case 2). The highest sensitivity is close to 55 dB/%, almost an order of magnitude higher than in the remaining cases. The lowest sensitivity of the system is also no smaller than 1 dB/%.

**Figure 9:**
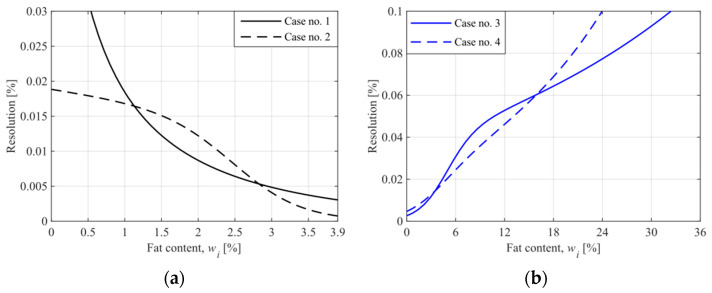
The resolution curves of the proposed system for milk samples ((**a**)—cases no. 1 and 2, for the operating point at 3.9%, (**b**)—cases no. 3 and 4, for the operating point at 0%).

Another critical parameter of a measuring instrument is resolution. In this study, the resolution is defined as the slightest detectable incremental change of the input parameter specified. The resolution of measured content depends on the A/D converter resolution (i.e., number of bits) used to measure the detector’s output voltage. We could detect the smallest possible increment of 0.81 mV at 12 bits with the reference voltage set at 3.3 V. Upon taking the slope of the power-voltage conversion curve of the detector, which was estimated at 18.88 mV/dB, the resolution in terms of output power can be determined using the following relationship:

(4) $$\Delta P=\frac{\Delta U}{C_{d}}$$

where ΔP is the increment of output power in dB, ΔU is the increment output voltage in mV, and $C_{d}$ is the slope of the power-voltage conversion curve in mV/dB. The result obtained for Δ*P* was 0.043 dB. Finally, the resolution of the system was described as follows:

(5) $$R_{o}\left(w_{i}\right)=\frac{\Delta P}{\left|S\left(w_{i}\right)\right|}$$

with the unit of (%). The obtained resolution curves of the system for milk measurement are shown in Figure 9. These curves describe the theoretical resolution of the proposed method, i.e., the resolution in the presence of the quantization noise only.

**Figure 10:**
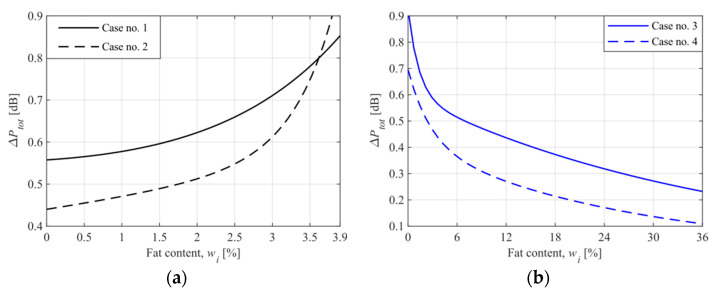
The total error of power measurement based on the standard deviation of power readings during calibration of the system ((**a**)—cases no. 1 and 2, for the operating point at 3.9%, (**b**)—cases no. 3 and 4, for the operating point at 0%).

In practice, due to system noise that is significantly higher than the quantization noise, the system’s ability to distinguish two adjacent values of the concentration is lower. The system’s total noise is responsible for the spread of the measurements and contributes to the precision and accuracy of the system. The deviation in power measurements results mainly from the instability of the signal generator, where the frequency fluctuation and the power supply instability are essential. The total error of power measurement ($\Delta P_{tot}$) was estimated using the standard deviation of power readings during calibration of the system. The obtained error curves are shown in Figure 10.

**Figure 11:**
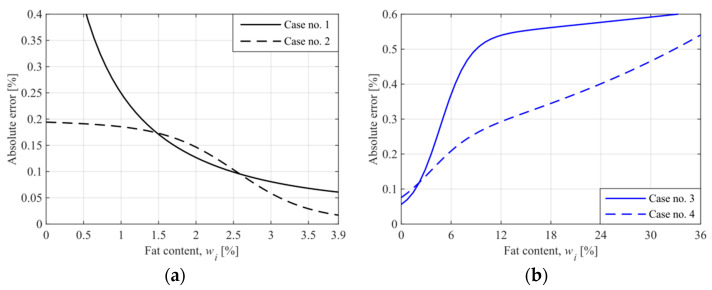
The absolute error of measurement for the milk samples ((**a**)—cases no. 1 and 2, for the operating point at 3.9%, (**b**)—cases no. 3 and 4, for the operating point at 0%).

As one can see, the power measurement error reaches the highest values near the system’s operating point. Fortunately, the fat content measurement error cannot be interpreted in the same way. To clarify this issue, we determined the final error curves. The obtained power error curves were approximated, and a function $\Delta P_{tot}\left(w_{i}\right)$ was obtained. Then, an analog of Equation (5) was used to write the following equation:

(6) $$\Delta w_{tot}\left(w_{i}\right)=\frac{\Delta P_{tot}\left(w_{i}\right)}{\left|S\left(w_{i}\right)\right|}$$

where $\Delta w_{tot}$ is the absolute error of measurement for the milk samples. The obtained curves are shown in Figure 11.

It appears from Equation (6) that the higher sensitivity of the system for a given content $w_{i}$, the lower the absolute error of measurement. Therefore, the proposed system operates with the lowest error for Case 2 (the operating point at 3.9%), for which the ultrahigh sensitivity was obtained. In this case, the absolute error ranges from 0.017% to 0.194%.

The length of the microfluidic channel and the position of the operating point influences the error curve in different ways. There are, however, some features in common. The absolute error decreases near the operating point. By proper selection of the microchannel length, the error curve can be made almost flat in a wide range of values of the observed quantity (see Case 2 in Figure 11a for $w_{i}$ from 0 to 1.5).

Regarding the relative error of measurement, the problem of “zero measurements” arises. The relative error close to the operating point for Case 2 was only determined for demonstration purposes. For the milk fat content in the range of 3–3.9%, the relative error decreases from 1.95% to 0.43% (with the concentration measured in %). The value of 0.43% is comparable with the error specified by the industry standard MilkoScan FT infrared milk analyzer. This comparison proves that the proposed system can be exact and has considerable potential in selected applications, such as low-cost, hand-held gauges for personal use or as a precise sensor in control systems in the food or chemical industries. It also has considerable potential to be used in the communication equipment industry, developing and producing devices based on liquid dielectrics.

Finally, the precision and accuracy of the proposed system can be assessed. All obtained error curves (Figure 10) describe the system’s precision directly since the concentration of standard samples was directly mapped to the average readings of the power detector. The system’s accuracy depends on the accuracy of the standard samples’ specifications. Unfortunately, this accuracy was unknown, and hence the accuracy of the proposed system could not be determined.

## 6. Conclusions

In summary, the proposed system offers ultrahigh sensitivity up to 55 dB/%. However, this sensitivity is limited by the length of the microstrip line integrated with the microfluidic channel. Therefore, even higher sensitivity can be anticipated for a longer microstrip line.

It was shown that the critical features of the proposed system depend on the position of the operating point, which can be freely selected within the available range of the standard curve. Moreover, the system provides a wide measurement range with acceptable precision at the far end of the standard curve.

## Figures and Tables

**Table 1:** LTCC sensor modules basic dimensions (mm).

Type	*w*	*l*	*w_c_*	*h_c_*	*h_s_*	Length
Short channel (water)	20.00	53.70	2.24	1.00	1.50	~31.00
Long channel (water)	40.50	49.00	2.24	1.00	1.50	~83.00

## Data Availability

The study did not report any data.
